# Dexamethasone prevents TACE-induced adverse events: A meta-analysis

**DOI:** 10.1097/MD.0000000000023191

**Published:** 2020-11-20

**Authors:** Lei Chang, Wei Wang, Nanhui Jiang, Fengying Rao, Cheng Gong, Ping Wu, Jian Yang, Zhisu Liu, Tao Guo

**Affiliations:** a Department of Hepatobiliary and Pancreatic Surgery, Zhongnan Hospital of Wuhan University, Wuhan; b School of Nursing, Huanggang Polytechnic College, Huanggang; c Department of Intensive Care Unit, Zhongnan Hospital of Wuhan University, Wuhan; d Department of Pediatric Surgery, Guangzhou Institute of Pediatrics, Guangzhou Women and Children's Medical Center, Guangzhou Medical University, Guangzhou; e School of Basic Medical Sciences, Weifang Medical University, Weifang, China.

**Keywords:** dexamethasone, meta-analysis, transcatheter arterial chemoembolization

## Abstract

**Background:**

While dexamethasone has been applied following transcatheter arterial chemoembolization (TACE) for years, its clinical effects have not been determined. In the current study, we aimed to evaluate the efficacy of dexamethasone in preventing adverse events induced by TACE.

**Methods:**

Literature retrieval was conducted using globally recognized online databases, namely MEDLINE, EMBASE, and Cochrane Central, to identify randomized controlled trials (RCTs) of dexamethasone application in patients undergoing TACE. The relative odds ratios (ORs) of incidence rates of three adverse events, namely, fever, abdominal pain and nausea/vomiting, were calculated. The value of I^2^ was applied to evaluate the heterogeneity of the trials, and the overall publication bias was assessed with Egger test.

**Results:**

Four RCTs containing 350 subjects were included for the pooled estimation. Dexamethasone significantly reduced the incidence rate of TACE-induced adverse events (OR = 1.237, 95% CI: 1.170–1.308, *P* < .001) with moderate heterogeneity (I^2^ = 46.0%). The result of Egger test revealed a publication bias for the included studies.

**Conclusion:**

The current meta-analysis confirmed the efficacy of dexamethasone in preventing TACE-induced adverse events. To confirm the practicality of dexamethasone use with TACE, further studies with large sample sizes are warranted to update the evidence-based analyses.

## 1 Introduction

Liver cancer is one of the most common malignant tumors and has the fourth highest cancer-related mortality rate worldwide.^[1,2]^ Based on the developments of therapeutic procedures and further understanding of pathogenesis, the current treatments for liver cancer are multifarious and can be selected based on the characteristics of the tumor and systemic status of the patient.^[3]^ In addition to surgical resection and traditional chemoradiotherapy, interventional therapy, such as transcatheter arterial chemoembolization (TACE), is widely performed in patients suffering from liver cancer.

TACE is recognized as an efficient and safe therapeutic procedure for unresectable multifocal and massive hepatocellular carcinoma (HCC).^[4,5]^ By inducing ischemia and necrosis of tumor tissue through arterial chemoembolization, TACE efficiently suppresses the development of liver cancer and can be applied to recurrence, which occurs quite commonly in HCC; therefore, TACE contributes to tumor control and retreatment.^[6,7]^ Given its feasibility and safety, TACE has been utilized for liver cancer for decades; nevertheless, postembolization syndrome (PES), which refers to a series of typical symptoms including fever, abdominal pain, and nausea/vomiting generally induced by TACE, turns out to be a notable clinical issue related to prognosis.^[8]^ Presenting from 1 day to 5 days after the TACE procedure, PES was reported to result in prolonged postprocedural hospitalization and reduce the patients’ quality of life postoperatively.^[9,10]^ While the etiopathogenesis of PES has not yet been distinctly established, the current theory deems that it may be the systemic inflammation and adverse reactions of chemotherapeutics that contribute to the incidence of PES. Specifically, the inflammatory response is thought to be due to chemoembolization-induced ischemia, cytolysis, and/or necrotic hepatocytes.^[11]^

To inhibit the inflammatory response and alleviate clinical symptoms, a series of medications including antiemetic, analgesic and steroid medications are applied in post-TACE patients.^[12]^ As a steroid preparation, dexamethasone is known to have efficient anti-inflammation and immunosuppression effects. Dexamethasone has been proven to reduce the incidence of side effects induced by emetogenic chemotherapy.^[13]^ In recent years, some clinical trials have also been conducted to verify the practicability and safety of dexamethasone in preventing TACE-induced adverse events.^[14,15]^ To date, no comprehensive meta-evidence supports the benefits of dexamethasone for patients undergoing TACE. Therefore, we performed the current meta-analysis to provide quantitative evidence-based suggestions for the clinical utilization of dexamethasone to prevent PES induced by TACE.

## 2 Methods

### 2.1 Literature search and retrieval

Current meta-analysis was based entirely on previous published studies which had declared ethical approvals and no original clinical raw data was collected or utilized, thereby ethical approval was not conducted for this study. The current meta-analysis of the published studies of perioperative dexamethasone application for TACE-associated adverse events was conducted following the principle of the PRISMA statement.^[16]^ We registered the current study online with PROSPERO (ID CRD42020176322). To obtain access to the relevant trials, MeSH terms including ‘liver neoplasms’ ‘chemoembolization, therapeutic’ ‘infusion, intra-arterial’ and ‘dexamethasone’ were used separately and in combination to search the electronic databases recognized globally, including MEDLINE, EMBASE, and Cochrane Central. The studies of interest were initially screened by the inclusion criteria of full text with English abstracts; there were no limitations according to full-text language or publication date.

### 2.2 Inclusion and exclusion criteria

The study inclusion criteria were as follows:

(1) studies conducted on adult participants;
(2) studies on liver cancer patients undergoing TACE;
(3) randomized controlled trials (RCTs);
(4) application of dexamethasone as an experimental intervention; and
(5) available targeted parameters reported in the form of data.

The exclusion criteria were established as follows:

(1) non-RCTs;
(2) inadequate raw data of interest;
(3) replicated studies;
(4) basic science or animal experiment studies;
(5) study protocols, comments, reviews, case reports, or conference summaries.

### 2.3 Parametric data selection and extraction

In the current study, we evaluated the capability of dexamethasone to reduce TACE-associated adverse events. Considering the complicated connotation of adverse events, the 3 typical symptoms, namely, nausea and/or vomiting, fever and abdominal pain, were selected as the outcomes for this pooled quantitative analysis. The extraction of data was accomplished by 2 investigators who reviewed the full texts independently, and any debate was discussed within the group until a unanimous verdict was reached. The general information (author, publication year and country) and characteristics (study design, sample size, therapeutic regimen, observational duration and parameters) of the included studies were then itemized in a predesigned table. Focusing on the effects of dexamethasone, the form and dosage of medications were not considered.

### 2.4 Quality assessment

The quality assessment of the included trials was carried out independently by 2 investigators using the Cochrane Risk of Bias assessment tool.^[17]^ The relative risks of bias for individual trials was determined by the following items:

(1) selection bias;
(2) performance bias;
(3) detection bias;
(4) attrition bias;
(5) reporting bias and
(6) other bias.

The grades from the bias assessment were summarized in a graphical representation utilizing Review Manager software (version 5.3).

### 2.5 Statistical analysis

Pooling the results of individual studies together, the overall odds ratios (ORs) and their 95% confidence intervals (CIs) regarding the 3 main outcomes were determined to estimate the relative efficacy of dexamethasone compared with the control group. The I^2^ index was calculated to evaluate the heterogeneity among the included studies. A fixed effects model was used to estimate the overall ORs if I^2^ < 50%, which indicates that no significant heterogeneity exists. In contrast, a random effects model was applied when I^2^ > 50%.^[18]^ The interpretation of heterogeneity was roughly defined as follows: I^2^ values of 0% to 30% represented heterogeneity that might not be important; I^2^ values of 30% to 60% represented moderate heterogeneity; I^2^ values of 60% or more represented considerable heterogeneity. Moreover, Egger test was conducted to assess publication bias, and a value of *P* < .05 was identified as an indication of significant publication bias. The statistical manipulation and graphic rendering for this meta-analysis were accomplished utilizing the STATA software package (Version 15.0).

## 3 Results

### 3.1 Study characteristics and quality assessment

**Figure 1:**
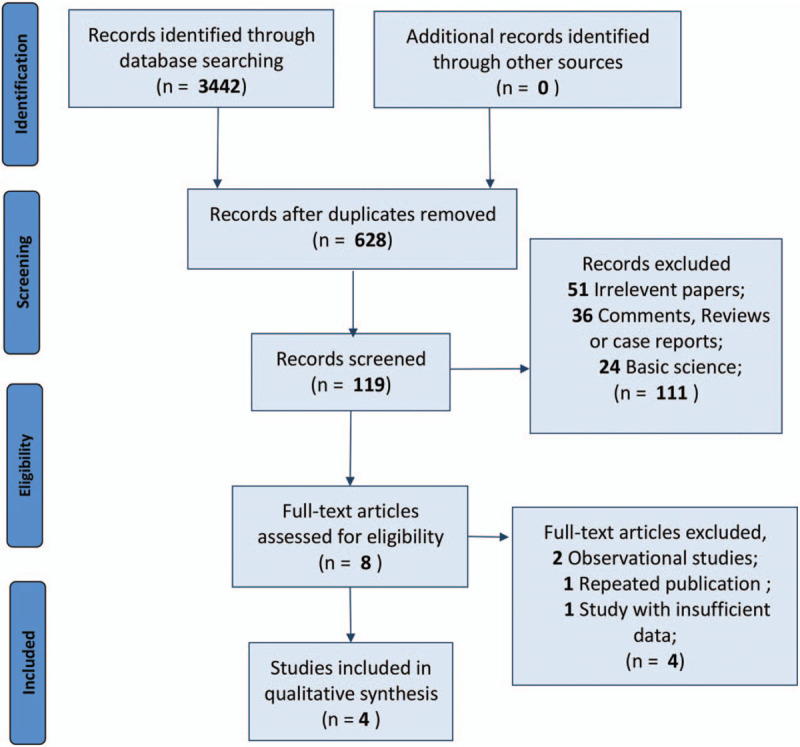
Flow diagram of the process of selecting studies for current meta-analysis.

After screening the 3442 papers obtained initially, 4 RCTs were identified to meet the inclusion criteria (Fig. 1).^[19–22]^ A total of 350 subjects were ultimately included in the pooled comparison. The 2 RCTs were conducted in China and contained multiple groups; thus, we compared the experimental groups that applied dexamethasone (alone or in combination with other ingredients) with the control group. The general information about the studies and the primary characteristics are listed in Table 1.

**Table 1:** Characteristics of included studies.

Author & Year	Country	Design	Sample size	Dexamethasone application	Observation	Parameter
Feng 2005 (1)	China	RCT	60	Oral administration (2.25 mg. bid.) from PRD 3 to POD 4	1 week after TACE	Nausea and vomiting; Fever; Abdominal pain
Feng 2005 (2)	China	RCT	(60)			Nausea and vomiting; Fever; Abdominal pain
Feng 2009 (1)	China	RCT	90	Oral administration (2.25 mg. bid.) from PRD 3 to POD 4	1 week after TACE	Nausea and vomiting; Fever; Abdominal pain
Feng 2009 (2)	China	RCT	(90)			Nausea and vomiting; Fever; Abdominal pain
Ogasawara 2017	Japan	RCT	119	Intravenous administration (20 mg. qd.) On POD 0; Intravenous administration (8 mg. qd.) On POD 1 and 2	6 days after TACE	Nausea; Vomiting; Fever; Abdominal pain
Yang 2017	Korea	RCT	81	Intravenous administration (12 mg) before TACE	48 hours after TACE	Nausea; Vomiting; Fever; Abdominal pain

POD = postoperative day, PRD = preoperative day, RCT = randomized controlled trial.

**Figure 2:**
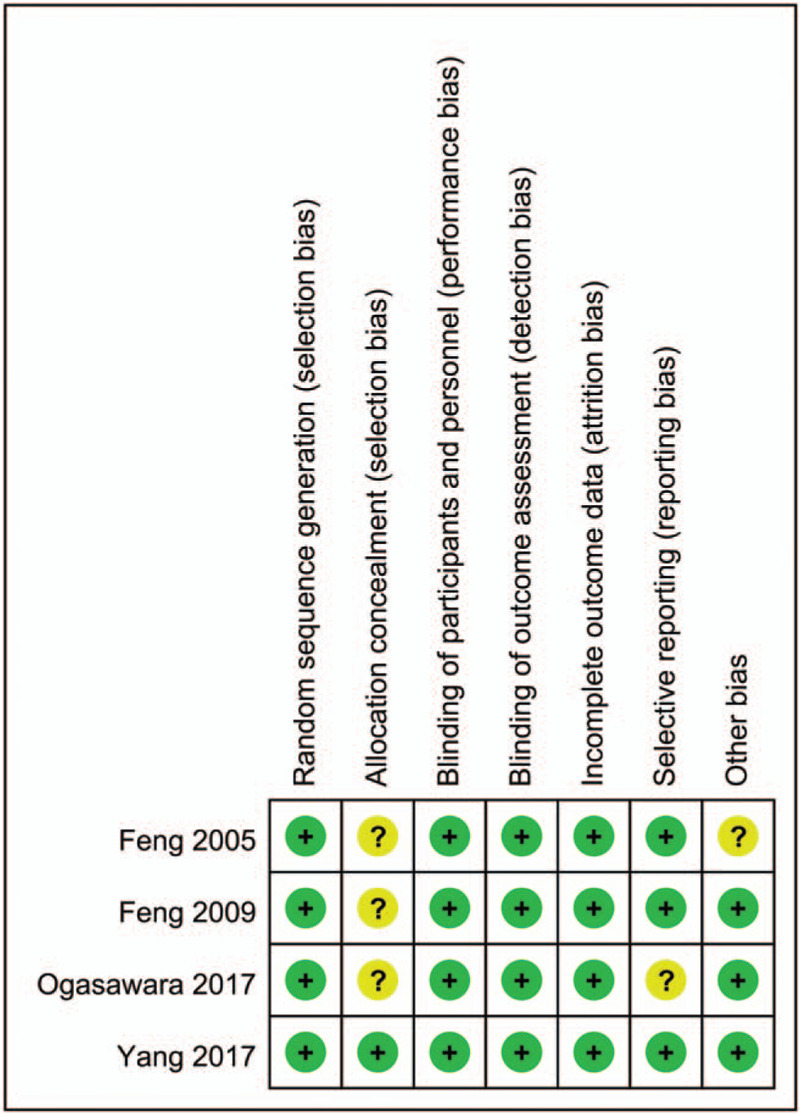
Bias assessment of each risk of bias item for each included study.

For the assessment of bias, an overall high quality of the 4 RCTs was recognized, as exhibited in Figure 2. All of the trials were designed to assign subjects by means of random sequence generation and utilized blinding methods in the intervention and outcome detection processes.

### 3.2 Dexamethasone significantly reduces adverse event after TACE

**Figure 3:**
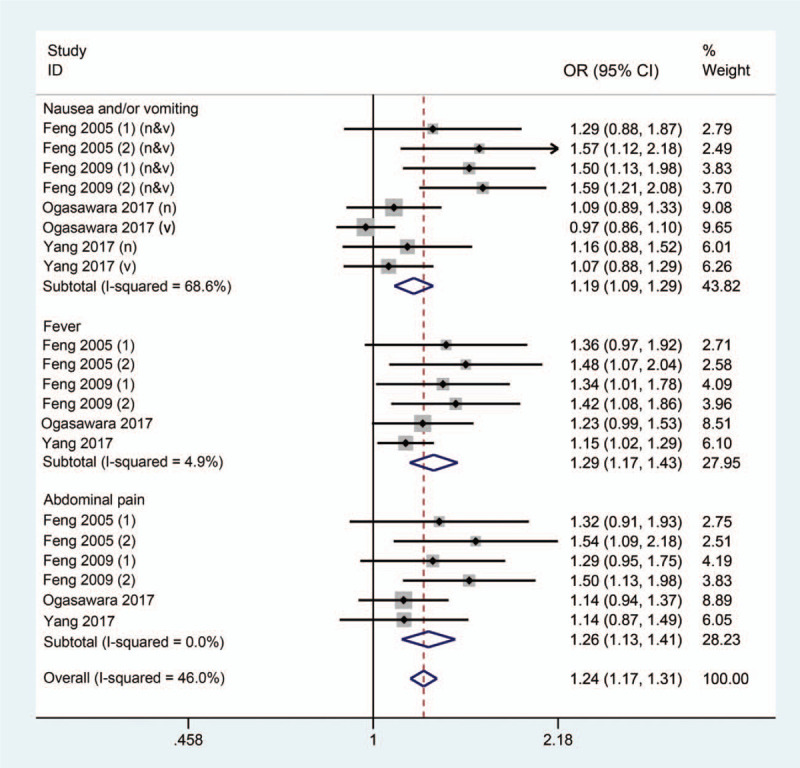
Forest plot comparing the dexamethasone and control groups with respect to adverse events. n, nausea; v, vomiting.

To evaluate the efficacy of dexamethasone on reducing TACE-associated adverse events, we performed the current quantitative synthesis of individual results based on a fixed effects model. The pooled results indicated that dexamethasone significantly reduced the incidence of adverse events after TACE (*P* < .001). The overall OR was 1.237 (95% CI: 1.170- 1.308) (Fig. 3). Additionally, the ORs specific to each of the 3 outcomes were calculated. The incidence rate of nausea/vomiting decreased with an OR of 1.187 (95% CI: 1.093–1.288, *P* < .001). Moreover, dexamethasone turned was associated with less risk of fever (OR = 1.291; 95% CI: 1.167–1.428; *P* < .001) and alleviation of postoperative abdominal pain (OR = 1.263; 95% CI: 1.170–1.308; *P* < .001). Regarding heterogeneity, the overall I^2^ of the main result indicated moderate heterogeneity (I^2^ = 46%). Specifically, high heterogeneity was observed in the nausea/vomiting outcome subgroup with I^2^ = 68.6%, while the other outcome subgroups for fever and pain revealed insignificant heterogeneity with I^2^ = 4.9% and I^2^ = 0%, respectively.

### 3.3 Publication bias

**Figure 4:**
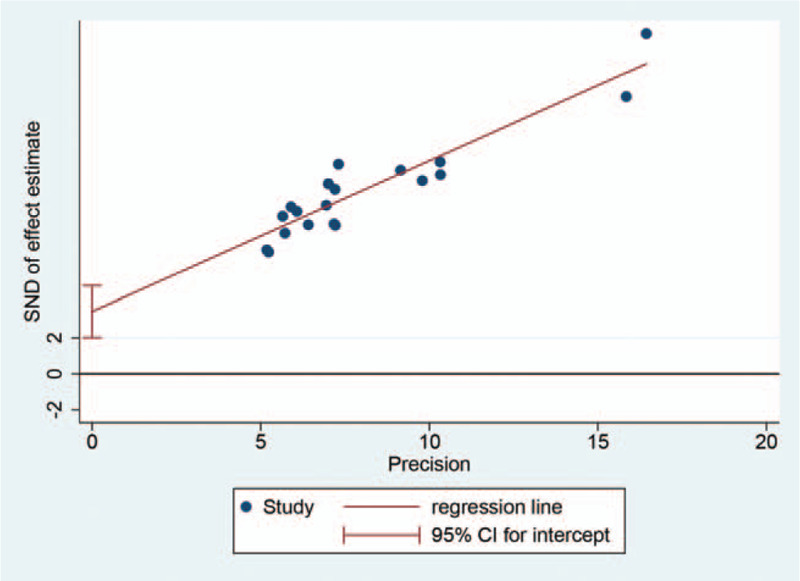
Egger publication bias plot of adverse events.

Egger test was applied to assess the overall publication bias, and the regression line is exhibited in Figure 4. The *P* value of bias was <0.05, implicating an obvious publication bias for the included studies.

## 4 Discussion

The current meta-analysis evaluated the efficacy of dexamethasone in preventing TACE-induced adverse events by pooling data from 4 RCTs. The pooled results revealed that the cumulative incidence rate of 3 outcomes, namely fever, abdominal pain and nausea/vomiting, was significantly reduced by dexamethasone. Furthermore, the effects of dexamethasone each individual outcome were consistent with the main result.

TACE, which was first proposed by Professor Yamada in 1978, has been utilized for unresectable and recurrent liver neoplasms for decades due to its feasibility and therapeutic safety. Due to improvements in radiography and anticarcinogenic medication, TACE technology has become recognized as a superior treatment in HCC worldwide. The rationale for TACE includes selective embolization with intra-arterial infusion of lipiodol and chemotherapeutic drugs, which then block the blood supply of the tumor, thus inducing the shrinkage and necrosis of tumor tissues.^[23,24]^ With increased concerns about the survival quality of patients suffering from HCC, the management of TACE-induced adverse events tends to be taken seriously. According to a systematic review of the efficacy and safety of TACE, approximately 47.7% of patients undergoing TACE were diagnosed with PES.^[10]^ In addition, the incidence rates of fever, abdominal pain and nausea/vomiting were highest among TACE-induced adverse events;^[10]^ these events were selected as the main outcomes in the current meta-analysis. The duration of hospitalization after TACE ranges from 12 hours to 6 days owing to individual and regional diversities,^[25–27]^ which also impacts differences in outcomes resulting from post-TACE management.

It is thought that the cytolysis and necrosis of tumor cells following chemoembolization may lead to inflammatory cytokine release and a systemic stress response. Simultaneously, intra-arterial infusion can cause ischemia and injury of normal hepatic tissues due to differences in the operation. Moreover, the invasive procedures and side effects of chemotherapeutic drugs inevitably influence the homeostasis of patients.^[14,20]^ Therefore, TACE-induced PES is mainly controlled with symptomatic treatments due to indeterminate etiopathogenesis at present. The application of a 5-HT3 receptor antagonist was proven to treat TACE-induced nausea and vomiting efficiently, and postoperative pain could be controlled by analgesic agents, such as oxycodone.^[28,29]^ Considering the metabolic load on the liver caused by multiple medications, dexamethasone, which has promising anti-inflammatory effects and inhibits immunoreactions, was expected to prevent PES. Several retrospective and prospective clinical trials have confirmed the function of dexamethasone in reducing TACE-induced adverse events.^[15,22]^ Targeting the glucocorticoid receptor dexamethasone plays an important role in inflammatory and immune responses through the genetic effects of inhibiting the expression of inflammatory mediators and inducing the apoptosis of immunocytes. Furthermore, the membrane-stabilizing action of dexamethasone has been recognized for a long time. In addition to the capability of maintaining lysosomal membrane integrity, dexamethasone could also regulate vascular permeability by strengthening cell-to-cell contacts.^[30,31]^ Based on the powerful effects of stabilizing the endothelium, dexamethasone plays an important role in both local and systemic inflammatory responses. Regarding antiemetic efficacy, it was reported that dexamethasone could prevent chemotherapy-induced cerebral nausea and vomiting,^[32]^ suggesting its potential in the management of PES. In the RCT by Ogasawara et al,^[21]^ prophylactic dexamethasone treatment improved post-TACE recovery and protected HCC patients from postoperative fever, anorexia, and nausea/vomiting. Moreover, the application of dexamethasone in patients with diabetes or impaired glucose tolerance maintained the beneficial effects, and there was no significant change in either hemoglobin A1c or glycol-albumin over the course of 12 weeks. For the subjects with current or prior hepatitis B virus (HBV) infection, no reactivation of HBV was induced by dexamethasone in the follow-up period.

To our knowledge the current meta-analysis demonstrated, for the first time, the clinical efficacy of dexamethasone in TACE based on quantitative analysis. We determined that dexamethasone could significantly ameliorate PES by decreasing nausea/vomiting, fever and pain. These results indicated that dexamethasone was multifunctional and could ameliorate a variety of adverse reactions from TACE. These results provide important clinical evidence for the guidance of patient management after the TACE procedure. More importantly, the current study not only provided an objective basis for dexamethasone application but also raised a new clinical research direction for the TACE procedure.

Nevertheless, we still admit some inevitable shortcomings of the current study. First, the included studies were conducted in Asia within a short span of time, and only a small number of relevant trials with small sample sizes were synthesized owing to our study design, hence the underlying local bias impacting our conclusions, which was confirmed by the result of Egger test assessing publication bias. In addition, the main results presented moderate overall heterogeneity (I^2^ = 46%). Specifically, this heterogeneity came from the nausea/vomiting outcome subgroup, which had substantial heterogeneity (I^2^ = 68.6%). We speculated that heterogeneity here was related to individual differences, medical backgrounds and/or the small number of included trials. Unfortunately, with only 4 RCTs included, a sensitivity analysis could not be conducted to assess these speculations. In addition, the adverse events induced by TACE, or the so-called PES, were not defined with determinate diagnostic criteria in the different trials. Therefore, we pooled the trials regarding 3 common symptoms after TACE to ensure quantitative consistency, while the safety of dexamethasone remained indistinct for the time being. Last, in spite of the aforementioned benefits of dexamethasone, it has been proposed that it might mask underlying postoperative infection and gastrointestinal hemorrhage, indicating an increased risk of missed diagnosis and delayed treatment.^[33]^ In the context of that proposal, our results suggest perioperative clinical benefits, but more high-quality clinical trials, especially trials with prolonged follow-up, are expected prior the recommendation of dexamethasone in clinical guidelines.

In conclusion, the results of this quantitative synthesis demonstrated that prophylactic dexamethasone treatment prevents adverse events induced by TACE. The current meta-analysis was an initial attempt to evaluate the efficacy of dexamethasone in patients undergoing TACE, providing an evidence-based suggestion and research direction for future studies on this topic. More high-quality clinical trials with large sample sizes and prolonged follow-up are expected to verify the safety and effects of dexamethasone with respect to the prevention of TACE-induced adverse events.

## Author contributions

Tao Guo and Lei Chang and Wei Wang designed the research; Tao Guo, Lei Chang, Wei Wang, Nanhui Jiang, Fengying Rao, Cheng Gong, Ping Wu, Jian Yang and Zhisu Liu performed the research and data collection; Lei Chang, Wei Wang contributed analytic tools and data analysis; Tao Guo and Lei Chang wrote the paper.
